# 24 km High-Performance Raman Distributed Temperature Sensing Using Low Water Peak Fiber and Optimized Denoising Neural Network

**DOI:** 10.3390/s22062139

**Published:** 2022-03-10

**Authors:** Hao Wu, Haoze Du, Can Zhao, Ming Tang

**Affiliations:** Wuhan National Laboratory for Optoelectronics, School of Optical and Electronic Information, Huazhong University of Science and Technology, Wuhan 430074, China; d202180843@hust.edu.cn (H.D.); zhao_can@hust.edu.cn (C.Z.); tangming@hust.edu.cn (M.T.)

**Keywords:** distributed temperature sensing, ROTDR, low water peak fiber, neural network

## Abstract

Raman distributed optical fiber temperature sensing (RDTS) has been extensively studied for decades because it enables accurate temperature measurements over long distances. The signal-to-noise ratio (SNR) is the main factor limiting the sensing distance and temperature accuracy of RDTS. We manufacture a low water peak optical fiber (LWPF) with low transmission loss to improve the SNR for long-distance application. Additionally, an optimized denoising neural network algorithm is developed to reduce noise and improve temperature accuracy. Finally, a maximum temperature uncertainty of 1.77 °C is achieved over a 24 km LWPF with a 1 m spatial resolution and a 1 s averaging time.

## 1. Introduction

Since the 1970s, with the birth of low-loss optical fibers and the wide application of lasers, research on optical fibers has been greatly expanded [1]. Due to its distinct advantages, distributed optical fiber temperature sensing has been extensively researched and implemented. First, the low-loss transmission of the fiber allows for long along-line temperature measurements over a long distance. Secondly, the main material of the optical fiber is silica, which gives the system excellent anti-electromagnetic interference properties, as well as resilience to high temperatures and corrosive environments. Furthermore, the fiber’s shape and flexibility make it useful in applications where space is restricted or portability is required. As a result, distributed optical fiber temperature sensing is widely employed in power grids, oil pipelines, nuclear power facilities, and other applications [2,3,4,5].

Distributed optical fiber temperature sensing obtains temperature change along with the optical fiber by measuring the scattered light of the injected pump light in the optical fiber. Scattering in optical fibers is divided into Rayleigh scattering, Brillouin scattering, and Raman scattering [6]. In 1981, Watson et al. found that Raman scattering spectra were mainly temperature-sensitive and remained almost unchanged under different pressures or applied strains [7]. Raman distributed temperature sensing (RDTS) becomes an appealing solution as a result of this property. The Raman-based optical time-domain reflectometer (ROTDR) is the main implementation of RDTS. By measuring the intensity of the anti-Stokes (AS) component of Raman scattered light, the influence of outside temperature on the fiber can be obtained. Sensing distance, average time, temperature accuracy, and spatial resolution are the indicators used to evaluate the RDTS system’s performance. These indicators are interrelated, and the overall performance of the system is determined by signal-to-noise ratio (SNR). There are now several strategies for improving the SNR of RDTS, which can be split into three categories: pulse code modulation, denoising algorithms, and the usage of special fibers. Since pulse code modulation will increase the hardware cost of the system, the research on denoising algorithms and special fibers has received attention in recent years.

Utilizing special fibers as sensing fibers is one approach to enhance a system’s SNR. The sensing fibers often used in conventional RDTS systems are multi-mode fibers (MMF) [8]. Since MMF have a larger mode field diameter than single-mode fibers (SMF), the nonlinear threshold of MMF is higher than of SMF. Therefore, MMF can allow higher input power than SMF, resulting in a larger scattering signal and a higher SNR. However, the modal dispersion of MMF makes the spatial resolution deteriorate for long-distance application. In 2017, Wang et al. proposed a quasi-single-mode few-mode fiber (FMF) RDTS system to reduce the dispersion effect. A 20 km RDTS with a 3 m spatial resolution, 6 °C temperature accuracy, and an 80 s average time was achieved [9]. In 2018, Liu et al. designed and fabricated a graded-index few-mode fiber (GI-FMF), which achieved a temperature resolution of 1 °C at 25 km, with a spatial resolution of 1.13 m and an average time of 90 s [10]. In 2021, Yang et al. increased temperature uncertainty to 0.5 °C with single-mode Raman gain fiber (RGF), with the system parameters set to a sensing distance of 2.9 km, a spatial resolution of 3 m, and a measurement time of 15 s [11]. These special fibers improve the SNR by increasing the input power or the Raman scattering coefficient. However, they did not optimize the transmission loss for Raman scattering, making the SNR decrease rapidly with fiber length. In common optical fiber, due to the influence of hydroxide ions during the fiber drawing process, the fiber attenuation will increase around a wavelength of 1383 nm, and the fiber attenuation spectrum shows an absorption peak, commonly known as the water peak [12]. To overcome this attenuation, the low water peak fiber (LWPF) is proposed. It has a relatively low loss for 1360–1460 nm light [13]. As the AS Raman scattering is about 1450 nm, the LWPF may improve the performance of RDTS.

On the other hand, improving the SNR can also be achieved by performing denoising processing on the obtained Raman scattering signals. Many methods, such as wavelet denoising (WD) algorithms and non-local mean algorithms, have been presented to enhance the SNR [14,15]. In recent years, with extensive research on deep learning, artificial neural network algorithms have also been proposed for RDTS denoising. In 2018, Wu et al. proposed a general residual convolutional image denoiser trained to denoise stacked Raman raw data traces [16]. In 2021, Zhang et al. employed the 1DDCNN model to denoise the distributed Raman backscattered raw data, and the temperature accuracy of the SMF-RDTS system was raised to 0.7 °C. Sensing distance was 10 km, with 3 m spatial resolution and a 1 s average time [17].

In this paper, we propose and experimentally demonstrate an RDTS system based on a single-mode LWPF. We designed and manufactured the single-mode LWPF with a low transmission loss of 0.21 dB/km for anti-Stokes (AS) Raman scattering light. To further improve the SNR of the system, we obtained a well-performing neural network by designing a training set with real noise. By denoising the AS curve with the optimized neural network, an LWPF RDTS system with high temperature accuracy and a long sensing distance is realized.

## 2. Fundamentals of the System

### 2.1. Raman Optical Time-Domain Reflectometry

Optical time-domain reflectometry was proposed by Barnowski et al. in 1976 and is widely used to monitor the operation of optical communication links [18]. The principle of ROTDR is to use the time when the Raman backscattered light in the fiber returns to the detector to calculate the location where the scattering occurs. The pulsed light enters the sensing fiber through an optical circulator and continuously collides with the medium in the fiber during propagation to generate Raman backscattered light. The Raman backscattered light is received by a photodetector through the optical circulator. It is assumed that the distance between the location where Raman scattering occurs and the transmitting end is *L*. The time taken for the pulsed light from the laser to be detected by the photodetector is *t*. The corresponding pulse light has traveled a total distance of *2L*, so the position *L* where Raman scattering occurs can be expressed as [8]:

(1) L=ct/2n

where *c* is the propagation speed of light in vacuum, and *n* is the refractive index of the fiber.

### 2.2. Temperature Demodulation Based on Spontaneous Raman Scattering

The RDTS is based on spontaneous Raman scattering. Raman scattering is a phenomenon caused by the scattering of incident pump light due to molecular vibration. The scattered light whose frequency is shifted to lower frequencies relative to the incident photon is called the Stokes component, whereas the scattered light shifted to higher frequencies is called the AS component. The light intensity of AS light is related to the external ambient temperature. When the temperature changes, the AS light intensity will also change, so the temperature can be demodulated by detecting the light intensity of AS light. When the ambient temperature is *T*_0_, the AS light intensity received by the detector is [19]:

(2) $$P_{as}(T_{0})=P_{0}K_{as}S\nu_{as}^{4}N_{as}(T_{0})\times \exp[-(\alpha_{as}+\alpha_{0})L]$$

where *P*_0_ is the light intensity of the incident light, and *L* is the distance from the location where the scattering occurs to the incident end of the fiber. *K_as_* represents the scattering coefficient of AS light, which is related to the cross-sectional size of the scattering, and *S* is the scattering factor. *ν_as_* is the frequency of AS light. *α*_0_ and *α_as_* refer to the transmission attenuation coefficient of source light and AS light in the fiber, respectively. *N_as_* is the Bose–Einstein distribution factor, which is related to the temperature *T*, and the specific relationship is [19]:

(3) $$N_{as}(T)={\left[\exp(\frac{h\Delta \nu }{k_{B}T})-1\right]}^{-1}$$

where *h* is Planck’s constant, *k_B_* is Boltzmann’s constant, and Δ*ν* is Raman shift. When the external ambient temperature becomes *T*, the light intensity of the AS light becomes:

(4) $$P_{as}(T)=P_{0}K_{as}S\nu_{as}^{4}N_{as}(T)\times \exp[-(\alpha_{as}+\alpha_{0})L]$$

Dividing Equations (2) and (4):

(5) $$\frac{P_{as}(T)}{P_{as}(T_{0})}=\frac{N_{as}(T)}{N_{as}(T_{0})}=\frac{\exp(h\Delta \nu /k_{B}T_{0})-1}{\exp(h\Delta \nu /k_{B}T)-1}$$

As *h*, *k_B_*, and Δ*ν* are constants, when the scattering intensity at the *T*_0_ is known, the fiber temperature *T* can be calculated from the corresponding scattering intensity. The expression of *T* derived from Equation (5) is [20]:

(6) $$T=\frac{h\Delta \nu }{k_{B}\ln\left[\frac{\exp(h\Delta \nu /k_{B}T_{0})-1}{P_{as}(T)}P_{as}(T_{0})+1\right]}$$

Therefore, the temperature accuracy is mainly determined by the fiber temperature sensitivity and the SNR of the measured scattering signals. The fiber temperature sensitivity is related to the material of the optical fiber and the composition of the optical fiber cable. A higher fiber temperature sensitivity also improves the SNR of Raman scattering signals. When light travels through the fiber, the SNR decreases due to transmission attenuation. Therefore, a smaller fiber attenuation coefficient results in less degradation of temperature accuracy over long distances.

## 3. Experiments Setup and Results

### 3.1. LWPF-Based RDTS System

**Figure 1:**
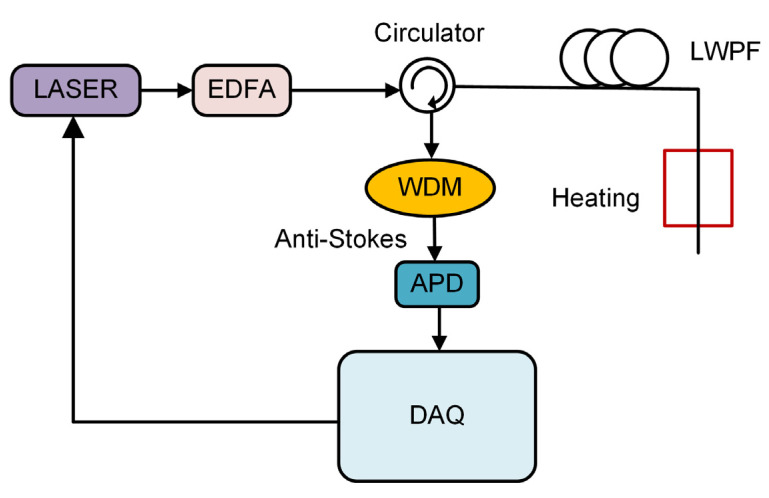
LWPF-based RDTS setup.

The LWPF-based RDTS setup is shown in Figure 1. An optical pulse with a wavelength of 1550 nm is generated through the laser with a pulse width of 10 ns. After being amplified by an erbium-doped fiber amplifier (EDFA), the optical pulse enters the LWPF through an optical circulator. The backscattered AS light enters port 2 of the circulator and is then output from port 3. A wavelength division multiplexer (WDM) is used to filter out the AS light. The AS light is then converted to an electrical signal through an avalanche photodetector (APD). The electrical signals are collected by a 250 MSa/s data acquisition card (DAQ) and averaged 4000 times in 1 s. A 24 km LWPF is used as a sensing fiber. The LWPF is manufactured by Yangtze Optical Fibre and Cable (YOFC) company and is designed to have low loss at 1450 nm. We fabricated the LWPF by a plasma chemical vapor deposition process. The base material was pure quartz, and the doping material was germanium tetrachloride. In the process, we prevented hydroxyl groups from entering the process equipment to reduce pollution, and surface etching treatment was used to remove surface pollutants and reduce the influence of residual hydroxyl pollutants.

**Figure 2:**
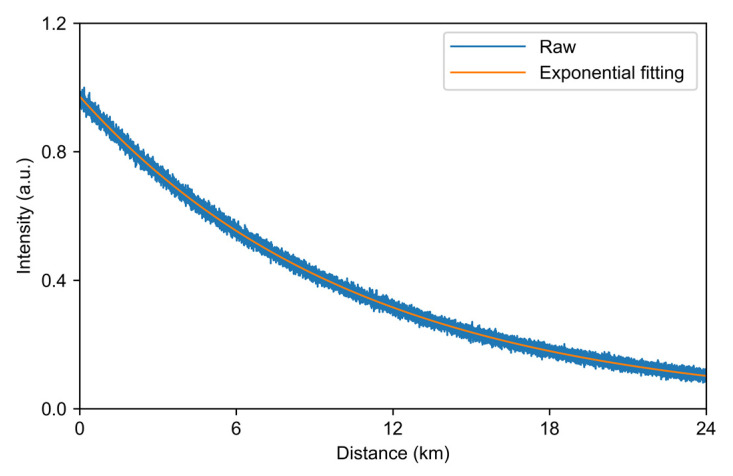
Distribution of AS signal.

The measured AS light intensity distribution is shown in Figure 2. The blue curve is the raw data of AS light, and the orange one is obtained after fitting the data. After the AS backscattered light passes through the 24 km LWPF, the light intensity drops by 9.78 dB, and the AS loss of the LWPF after conversion is about 0.4 dB/km. The loss of 1550 nm light in the fiber is approximately 0.19 dB/km, so the loss of Raman backscattered light in the fiber is 0.21 dB/km, whereas the loss of Raman backscattered light in a standard SMF is about 0.296 dB/km [11], and that in a standard MMF (OM2) is 0.3 dB/km [12]. Therefore, the LWPF has a relatively low transmission loss, resulting in a higher SNR for long-distance application.

**Figure 3:**
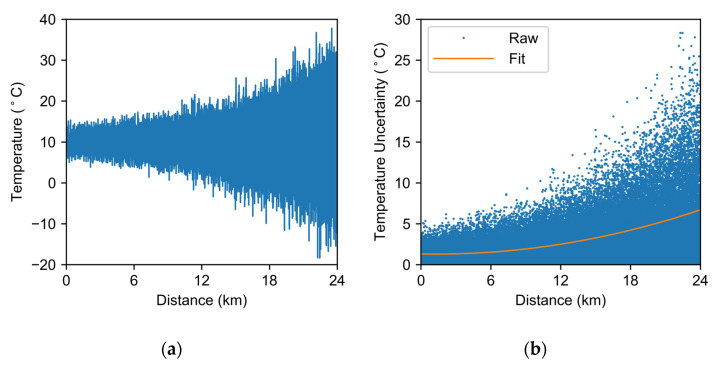
(**a**) Temperature profile measured with raw data at room temperature. (**b**) Temperature uncertainty distribution.

The temperature distribution curve obtained by the demodulation of the raw data is shown in Figure 3a. The temperature profile obtained for LWPF-based RDTS fluctuates at around 10 °C (room temperature). Due to the transmission loss, the measured temperature fluctuates more as the light travels through the fiber. The overall temperature uncertainty in the 24 km LWPF is shown in Figure 3b. The temperature uncertainty degrades as the sensing distance increases, reaching a maximum value of 6.69 °C at the fiber end.

### 3.2. LWPF-Based RDTS System with Denoising Neural Network

**Figure 4:**
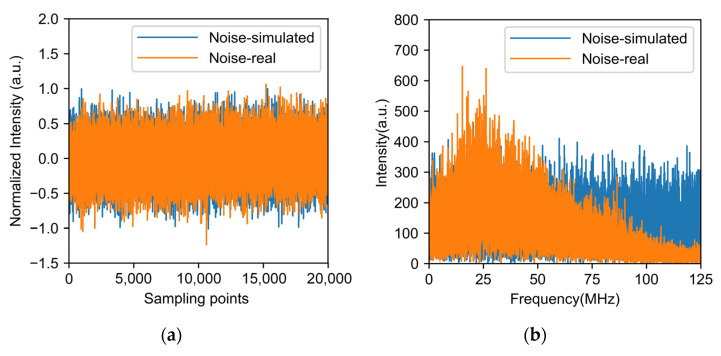
(**a**) Time domain and (**b**) frequency domain of simulated noise and real noise.

To further improve the performance of the system, we used an artificial neural network to reduce the noise. Using a neural network algorithm to denoise requires training data. The ideal training data are the real RDTS data and their corresponding noise-free data. However, noise is always present, and a completely noise-free signal cannot be obtained. Therefore, the simulation method is used to generate training data. In [17], the training set is constructed using a random function to generate white Gaussian noise to simulate the noise of the RDTS signal. However, the real noise is not the ideal Gaussian white noise. Although the noise-free signal is difficult to obtain, the real noise of the system can be easily measured by removing the sensing fiber and collecting the output of the photodetector. To investigate the influence of different noises, we designed two kinds of training sets. The first training set uses the white Gaussian noise generated by software simulation. The intensity-normalized curves are shown in Figure 4a in the time domain and the frequency domain in Figure 4b. The real noise is collected by our RDTS system and is shown in Figure 4. From the time-domain data in Figure 4a, the difference between the two kinds of noise is not obvious. However, from the frequency domain results in Figure 4b, the spectrum of simulated white noise is flat, whereas the spectrum of real noise is band-limited and is determined by the frequency response of the photodetector and the data acquisition card. When dealing with these two kinds of noise in the spectrum domain, different filters should be designed. Therefore, the neural network trained with simulated noise cannot achieve the best results on real data.

**Figure 5:**
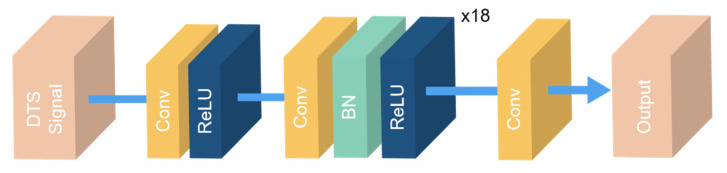
Neural network structure.

To study the effect of different noise types on neural network performance, we used the same neural network structure to analyze them. The neural network structure employed was the 1DDCNN proposed in [17], which shows much better denoising performance than conventional algorithms. As shown in Figure 5, the neural network is composed of convolution (Conv) layers [21], rectified linear units (ReLU) layers [22], and batch normalization (BN) layers [23]. The neural network consists of 20 Conv layers. The one-dimensional convolution kernel size is 3. The channel number of the first and last Conv layer is 1, and the channel number of the rest of the Conv layers is 64. The ReLU layers turn results less than 0 into 0, and the data greater than 0 remain unchanged, which brings nonlinearities. The BN is employed to normalize the data during training to help the network converge more quickly. The raw RDTS data are first normalized by dividing by its maximum value. Then, the normalized data are input into the neural network, and the denoised data are obtained through a layer-by-layer calculation.

**Figure 6:**
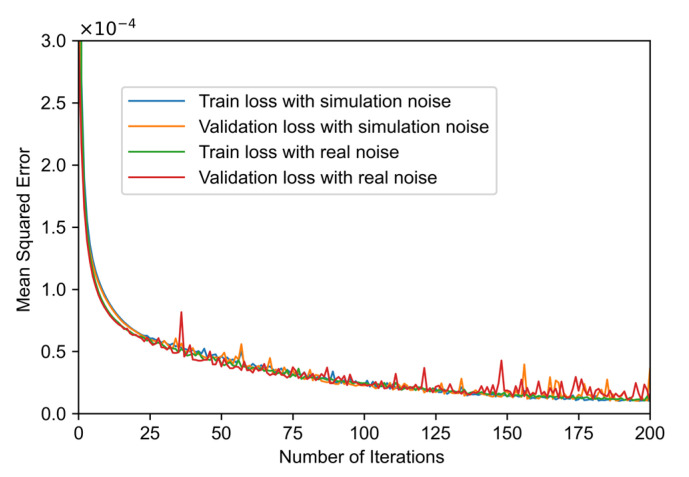
Variation of the mean square error with the number of iterations for training with simulated noise and real noise.

To train the neural network, we generated 10,000 curves for training with each of the two types of noise. Each curve contains 10,000 sample points. Of the data, 80% is used as the training set, and 20% is used as the validation set. As shown in Figure 6, after iteratively training for 200 epochs, the mean squared errors of both neural networks tend to be stable.

**Figure 7:**
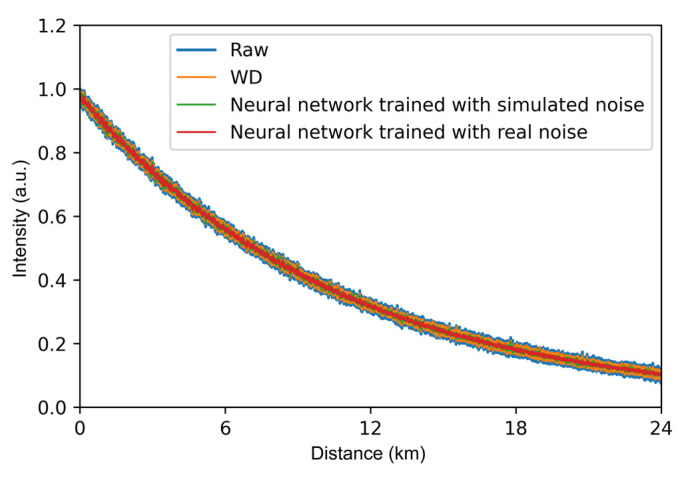
The raw AS signal and denoised results.

The AS light curves processed by the two neural networks are shown in Figure 7. Both neural networks can effectively reduce the noise of the distributed Raman scattering signal. As a comparison, we also show the result using WD, which is a conventional method for processing RDTS signals [14]. We used the db3 wavelet function to decompose the RDTS signal in three layers and the soft threshold method to process wavelet coefficients of each layer. Then, the processed wavelet coefficients and wavelet function are used to reconstruct the signal. The noise is reduced by 1.3 dB using the WD. Further, a noise reduction of 3.2 dB is achieved using the neural network based on simulated noise. The neural network based on actual noise can reduce noise by 4.1 dB.

**Figure 8:**
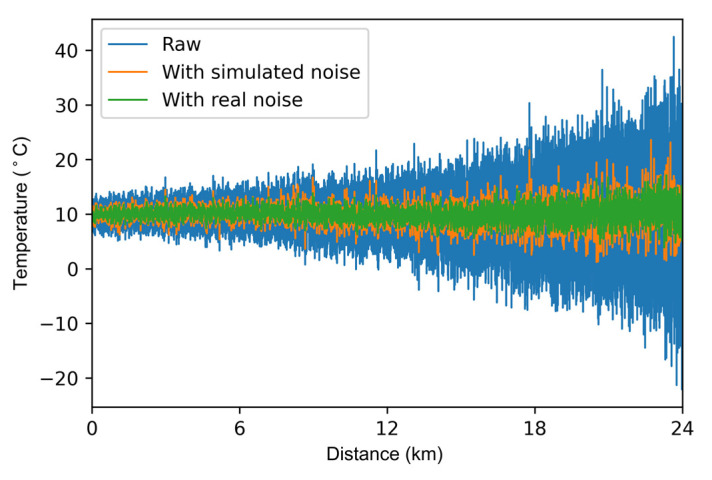
Temperature profile along with the fiber.

The temperature demodulation results are shown in Figure 8. The temperature profile processed by the neural network trained with the real noise has less fluctuation than the temperature profile processed by the neural network trained with the real noise.

**Figure 9:**
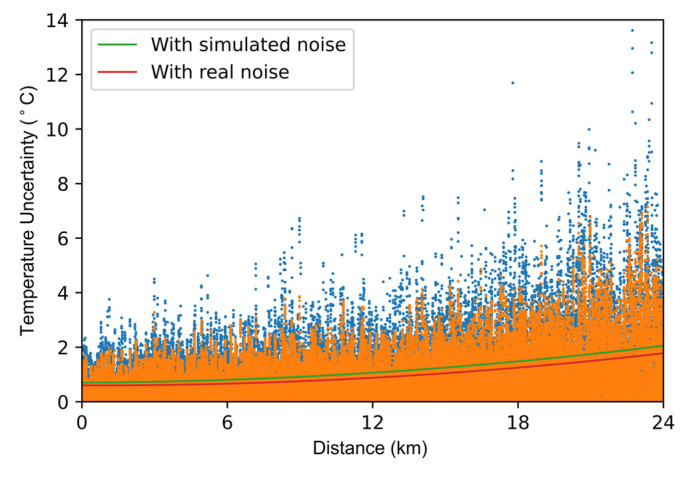
Distributed temperature uncertainty along fiber length.

The temperature uncertainty results are shown in Figure 9. After processed by the neural network with simulated noise, the temperature uncertainty is improved to 2.05 °C. Furthermore, the temperature uncertainty is 1.77 °C when using the neural network trained with real noise. The neural network trained with real noise can provide a better noise removal effect. Therefore, we process the RDTS data using the neural network trained with real noise in the following experiments.

**Figure 10:**
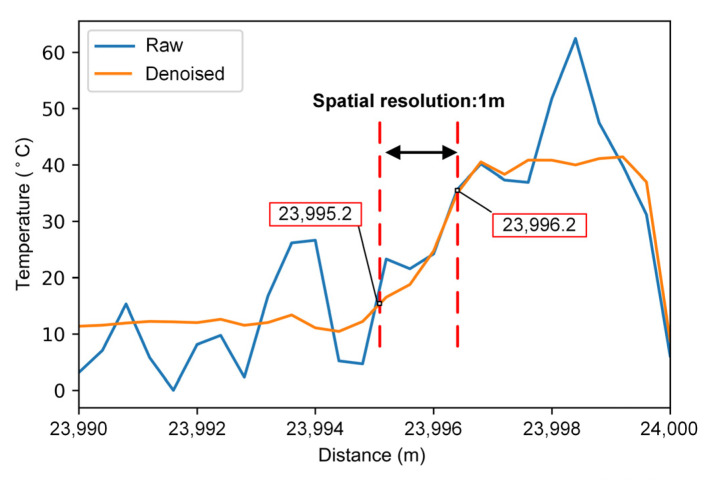
Temperature result when the end of 24 km LWPF heated.

Besides temperature accuracy, another important performance index of the RDTS system is spatial resolution. The spatial resolution of RDTS is generally defined as the fiber length corresponding to 10% to 90% of the rising edge of the temperature-varying signal. We placed about 2 m of fiber at the fiber end in a water bath to raise the temperature to 40 °C. The temperature curve of the corresponding region is shown in Figure 10. The absolute error of the unprocessed temperature curve in the heating section is 20 °C, and the temperature error after being processed by the neural network is less than 2 °C. As marked in Figure 10, the spatial resolution of the RDTS system remains 1 m over 24 km transmission.

### 3.3. Results with Different System Parameters

**Figure 11:**
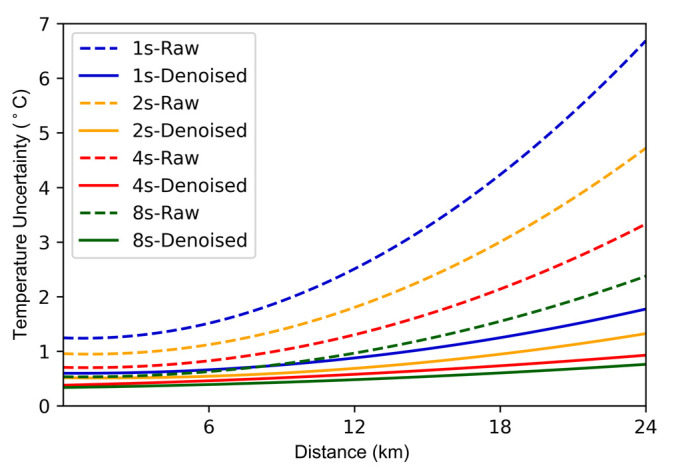
Temperature uncertainty with different spatial resolutions.

To illustrate the effectiveness of the denoising neural network on RDTS with different parameters, we set the pulse width of the laser to 10 ns, 20 ns, 40 ns, and 80 ns, corresponding to the spatial resolutions of 1 m, 2 m, 4 m, and 8 m. The temperature uncertainty curve is shown in Figure 11. The temperature uncertainty after denoising is significantly better than the unprocessed one. As the spatial resolution increases, the temperature accuracy is further optimized. When the spatial resolution is 8 m, the maximum temperature uncertainty after denoising can reach 0.91 °C.

**Figure 12:**
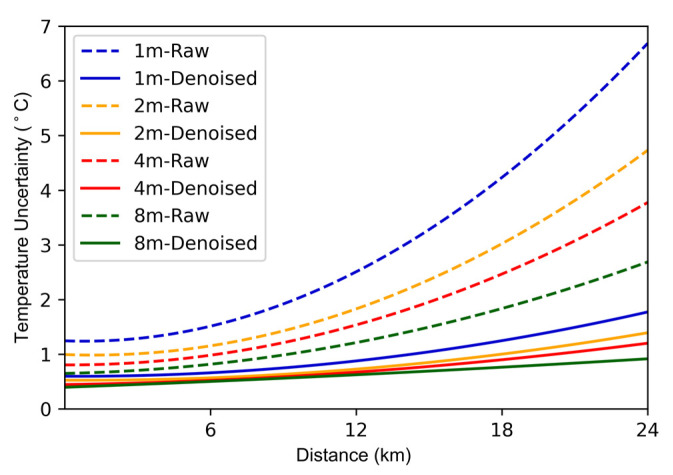
Temperature uncertainty with different averaging times.

In addition to pulse width, we also explored the effect of averaging time. The average time is set to 1 s, 2 s, 4 s, and 8 s, respectively. The temperature uncertainty curves of the raw and denoised data are shown in Figure 12. The temperature accuracy gradually increases as the averaging time increases. The temperature uncertainty obtained after 8 s average time with neural network processing reaches 0.76 °C.

## 4. Conclusions

We have proposed and experimentally demonstrated a high-performance LWPF with an optimized denoising neural network. Through special design, we have fabricated an LWPF with an AS light attenuation of only 0.21 dB/km at 1450 nm. Thus, the sensing range has been extended to 24 km. Furthermore, we trained an optimized denoising neural network using the collected noise as the training set. This neural network was then used to denoise the raw data of the LWPF-based RDTS. Finally, a 24 km LWPF-RDTS system was realized with a spatial resolution of 1 m, an average time of 1 s, and a temperature accuracy of 1.77 °C. In addition, increasing the averaging time or the pulse width can further improve the temperature accuracy. The scheme is simple in structure, low in cost, excellent in performance, and easy to deploy. It can play a good role in practical application scenarios.

## Data Availability

Not applicable.
